# Detailed Clinical Features of *PTPRQ*-Associated Hearing Loss Identified in a Large Japanese Hearing Loss Cohort

**DOI:** 10.3390/genes15040489

**Published:** 2024-04-12

**Authors:** Naoko Sakuma, Shin-ya Nishio, Shin-ichi Goto, Yohei Honkura, Kiyoshi Oda, Hidehiko Takeda, Marina Kobayashi, Kozo Kumakawa, Satoshi Iwasaki, Masahiro Takahashi, Taku Ito, Yasuhiro Arai, Yasuhiro Isono, Natsuko Obara, Takeshi Matsunobu, Kimihiro Okubo, Shin-ichi Usami

**Affiliations:** 1 Department of Otorhinolaryngology, Head and Neck Surgery, Nippon Medical School, Tokyo 113-8603, Japan; naoko-sakuma@nms.ac.jp (N.S.); t-matsunobu@nms.ac.jp (T.M.); ent-kimi@nms.ac.jp (K.O.); 2 Department of Hearing Implant Sciences, Shinshu University School of Medicine, 3-1-1, Asahi, Matsumoto City 390-8621, Japan; nishio@shinshu-u.ac.jp; 3 Department of Otorhinolaryngology, Hirosaki University Graduate School of Medicine, Hirosaki 036-8560, Japan; goto-s@hirosaki-u.ac.jp; 4 Department of Otolaryngology-Head and Neck Surgery, Tohoku University School of Medicine, Sendai 980-8575, Japan; y-honkura@orl.med.tohoku.ac.jp; 5 Department of Otolaryngology, Tohoku Rosai Hospital, Sendai 981-8563, Japan; oda.kiy@gmail.com; 6 Department of Otorhinolaryngology, Toranomon Hospital, Tokyo 105-8470, Japan; hidehiko.takeda@gmail.com (H.T.); marinakobayashi@nms.ac.jp (M.K.); 7 Department of Otolaryngology, Kamio Memorial Hospital, Tokyo 101-0063, Japan; kozo3000@hotmail.com; 8 Akasaka Toranomon Clinic, Tokyo 107-0052, Japan; 9 Department of Otorhinolaryngology, International University of Health and Welfare, Mita Hospital, Tokyo 108-8329, Japan; iwasakis@iuhw.ac.jp (S.I.); masa12_1@iuhw.ac.jp (M.T.); 10 Department of Otorhinolaryngology, Tokyo Medical and Dental University, Tokyo 113-8510, Japan; taku.oto@tmd.ac.jp; 11 Department of Otorhinolaryngology-Head and Neck Surgery, Yokohama City University School of Medicine, Yokohama 236-0004, Japan; arachan19775245@yahoo.co.jp; 12 Department of Otolaryngology, Yokohama City University Medical Center, Yokohama 232-0024, Japan; y_isono@isono-ent.com; 13 Department of Otolaryngology, Gifu University Graduate School of Medicine, Gifu City 501-1194, Japan; obaue926@icloud.com

**Keywords:** *PTPRQ*, non-syndromic hearing loss, DFNA73, DFNB84, congenital onset hearing loss, childhood onset hearing loss, progressive hearing loss, cochlear implantation

## Abstract

The *PTPRQ* gene has been identified as one of the genes responsible for non-syndromic sensorineural hearing loss (SNHL), and assigned as DFNA73 and DFNB84. To date, about 30 causative *PTPRQ* variants have been reported to cause SNHL. However, the detailed clinical features of *PTPRQ*-associated hearing loss (HL) remain unclear. In this study, 15,684 patients with SNHL were enrolled and genetic analysis was performed using massively parallel DNA sequencing (MPS) for 63 target deafness genes. We identified 17 possibly disease-causing *PTPRQ* variants in 13 Japanese patients, with 15 of the 17 variants regarded as novel. The majority of variants identified in this study were loss of function. Patients with *PTPRQ*-associated HL mostly showed congenital or childhood onset. Their hearing levels at high frequency deteriorated earlier than that at low frequency. The severity of HL progressed from moderate to severe or profound HL. Five patients with profound or severe HL received cochlear implantation, and the postoperative sound field threshold levels and discrimination scores were favorable. These findings will contribute to a greater understanding of the clinical features of *PTPRQ*-associated HL and may be relevant in clinical practice.

## 1. Introduction

Hearing loss (HL) is a common sensory impairment in humans, with the most common cause of congenital sensorineural hearing loss (SNHL) being genetic factors [1]. Genetic HL shows extreme heterogeneity, and non-syndromic hearing loss (HL) is reported to be responsible for approximately 75% of genetic HL. At present, 124 non-syndromic HL genes are listed on the Hereditary Hearing Loss homepage [2].

In Japan, genetic analysis using next-generation sequencing for HL is partially covered by social health insurance, and such genetic analysis has been widely used as a diagnostic tool recently. Therefore, many genetic variants have been identified in Japanese HL patients. The *PTPRQ* gene encodes protein tyrosine phosphatase receptor Q, and is one of the genes causing non-syndromic SNHL, assigned DFNA73 and DFNB84 [2]. The *PTPRQ* locus on chromosome 12q21.31 comprises 45 exons [3]. Previous studies revealed that the *PTPRQ* protein is localized in the basal region of the stereocilia in hair cells, and is required for stereocilia shaft connector formation and cochlear hair bundle maturation [4,5,6]. *PTPRQ* is a relatively rare causative gene for SNHL. To date, about 30 causative *PTPRQ* variants have been reported to cause SNHL [7]. However, the detailed clinical features of *PTPRQ*-associated HL remain unclear. Further, there have been few reports on the effectiveness of hearing aids or cochlear implants. In this study, we describe the detailed clinical findings, including the outcome of cochlear implantation, of Japanese patients with *PTPRQ* variants.

## 2. Materials and Methods

### 2.1. Subjects

A total of 15,684 patients with SNHL were enrolled in this study and genetic analysis was performed in the Department of Hearing Implant Sciences, Shinshu University School of Medicine. Among the 15,684 patients, 15 were identified with biallelic disease-causing *PTPRQ* variants. This study was approved by the respective ethical committees of the Shinshu University Ethical Committee (Approval number: No. 387—14 September 2012, No. 576—2 May 2017 and No. 718—7 March 2022) and the other participating institutions, and was conducted in accordance with the Declaration of Helsinki. Informed consent was obtained from all patients, or guardians in the case of minors.

### 2.2. Clinical Evaluations

Clinical information and peripheral blood or saliva samples were obtained from each proband and from all consenting relatives. Clinical information including (1) onset age; (2) hearing level and severity; (3) progression of HL; (4) pedigree; (5) intervention for HL and sound field threshold levels, with hearing aid or cochlear implant, were collected from medical charts. During the recruitment process, all participants were asked about any episodes or symptoms of vertigo. Evaluation of HL was performed by pure-tone audiometry. The average hearing level was calculated from the audiometric thresholds at four frequencies (500, 1000, 2000, and 4000 Hz). The severity of HL was classified based on the better hearing ear as normal (<20 dB), mild HL (21–40 dB), moderate HL (41–70 dB), severe HL (71–95 dB), and profound HL (>95 dB) [8]. The audiometric configurations were categorized into flat, low-frequency ascending, mid-frequency U-shaped, high-frequency gently sloping, and high-frequency steeply sloping, as reported previously [9]

### 2.3. Genetic Analysis

Massively parallel DNA sequencing (MPS) analysis for 63 target deafness genes was performed for all patients. The detailed protocol is described elsewhere [10]. In brief, an Ion AmpliSeq Custom Panel (ThermoFisher Scientific, Waltham, MA, USA) was designed using an Ion AmpliSeq Designer, and the amplicon libraries were prepared using an Ion AmpliSeq library kit version 2.0 (ThermoFisher Scientific, Waltham, MA, USA). The emulsion PCR and MPS were performed using an Ion PGM, Ion Proton or IonS5 sequencer (ThermoFisher Scientific, Waltham, MA, USA), and the sequence data were mapped against the human genome sequence (build GRCh37/hg19).

The protein-affecting variants (including the missense, nonsense, insertion/deletion, and splicing variants) with an allele frequency of less than 1% of the 1000 genome database [11], the 6500 exome variants, The Genome Aggregation Database [12], the human genetic variation database (dataset for 1208 Japanese exome variants) [13], the 38,000 Japanese genome variation database [14], and the 333 in-house Japanese normal hearing controls were selected. The annotation for each variant was analyzed by ANNOVAR software ver. 20191024 [15]. Functional in silico predictions were performed for missense variants by SIFT [16], PolyPhen2 [17], Mutation Taster [18], Mutation Assessor [19], FATHMM [20], and Combined Annotation Dependent Depletion (CADD) [21] software programs included in dbNSFP ver.3.5. The remaining *PTPRQ* variants were confirmed by direct sequencing. Segregation analysis for family members was also performed by direct sequencing.

The pathogenicity of the identified variants was evaluated using the American College of Medical Genetics (ACMG) standards and guidelines [22]. The variants previously reported as “Pathogenic” or “Likely Pathogenic” were applied the same pathogenicity classification, in cases where no contradictory evidence was identified. The variants classified as “Likely Pathogenic” or “Pathogenic” in ACMG standards and guidelines were considered to be causative variants. In addition, variants classified as being of “Uncertain Significance” were also considered to be causative, if all three of the following conditions were satisfied: (1) no other candidate variants were identified in the other 67 genes; (2) the allele frequency was extremely low in the control populations in ExAC03, gnomAD, ToMMo 54KJPN, and in-house controls; and (3) the CADD score was 20 or more.

## 3. Results

### 3.1. Identified Variations

In this study, we identified 17 possibly disease-causing *PTPRQ* variants in 13 Japanese HL patients. As shown in Table 1 and Table 2, six variants were nonsense variants, four variants were small insertions or deletions leading to frameshift change, one variant was an in-frame small deletion, four variants were splicing site variants, and two variants were missense variants. All 17 variants were located between exon 3 and exon 39. No variant was identified in exon 45, in which a variant reported to be causative for autosomal dominant HL (DFNA73) is located [23]. Two variants were already reported as pathogenic in previous reports. Of the remaining 15 novel variants, 13 variants were loss of function variants (including nonsense variants, small insertions or deletions, and splice site variants) and were classified as “Pathogenic” variants or “Likely Pathogenic” variants according to the ACMG guidelines [22]. Therefore, we concluded that those 13 variants cause *PTPRQ*-associated HL. The remaining one missense and one small in-frame deletion variants were classified as variants of “Uncertain Significance” under the ACMG guidelines [22]. However, we treated those variants as candidate variants for *PTPRQ*-associated HL as they fulfilled the criteria described in the Methods section. However, further studies are needed to confirm our conclusions regarding those variants.

All *PTPRQ*-associated HL patients identified in this study were unrelated. Three patients had homozygous variants, and 10 patients had compound heterozygous variants. *PTPRQ* variants have been reported as causative for autosomal recessive and dominant HL, and assigned as DFNB84 and DFNA73 [2]. However, all patients identified in this study carried biallelic variants and appeared to demonstrate autosomal recessive inheritance.

### 3.2. Clinical Features of Patients and Outcomes of Hearing Devices

**Figure 1:**
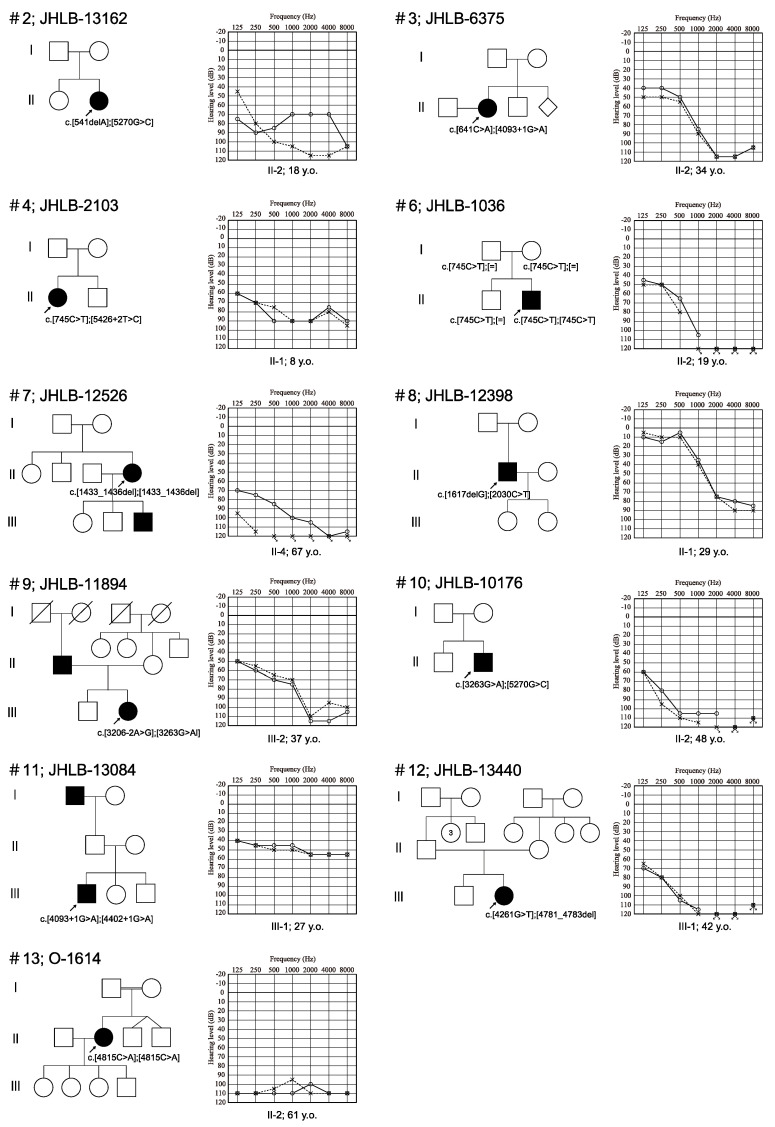
Pedigree and audiograms for each family for the *PTPRQ*-associated hearing loss patients in this study. Arrows show the probands in each family. The variants identified in this study are indicated on the figure.

The clinical characteristics of the *PTPRQ*-associated HL patients identified in this study are summarized in Table 1 and Figure 1. Four patients were male and eight patients were female. The mode of inheritance was sporadic in 11 patients and autosomal dominant in one patient. Regarding the onset age of the HL, five patients had congenital HL, two patients had prelingual onset HL (below the age of 6), and four patients had post-lingual onset HL (between the ages of 6 and 13). Nobody had adulthood onset HL. The hearing levels at the examination in the 12 patients varied from moderate to profound. Five and four patients had profound and severe HL, respectively, and three patients had moderate HL. All patients had bilateral HL. The audiometric configurations of the 12 patients for whom audiometric configuration information was available were categorized as flat type HL in eight and high-frequency steeply sloping type HL in four patients. All 11 patients had progressive HL. There were no individuals in whom HL did not deteriorate with time. Among the 13 *PTPRQ*-associated HL patients, four patients wore hearing aids (HAs) and five patients received cochlear implantation (CI). Three patients had symptoms of dizziness or vertigo, while seven patients had no symptoms (Table 1).

**Figure 2:**
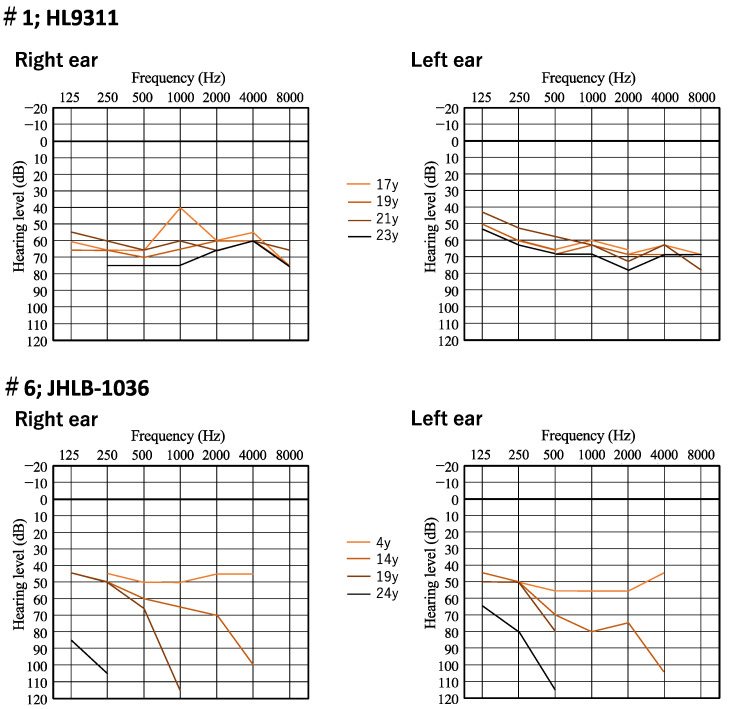
Serial audiograms of two patients with *PTPRQ* variants (family numbers 1 and 6).

**Figure 3:**
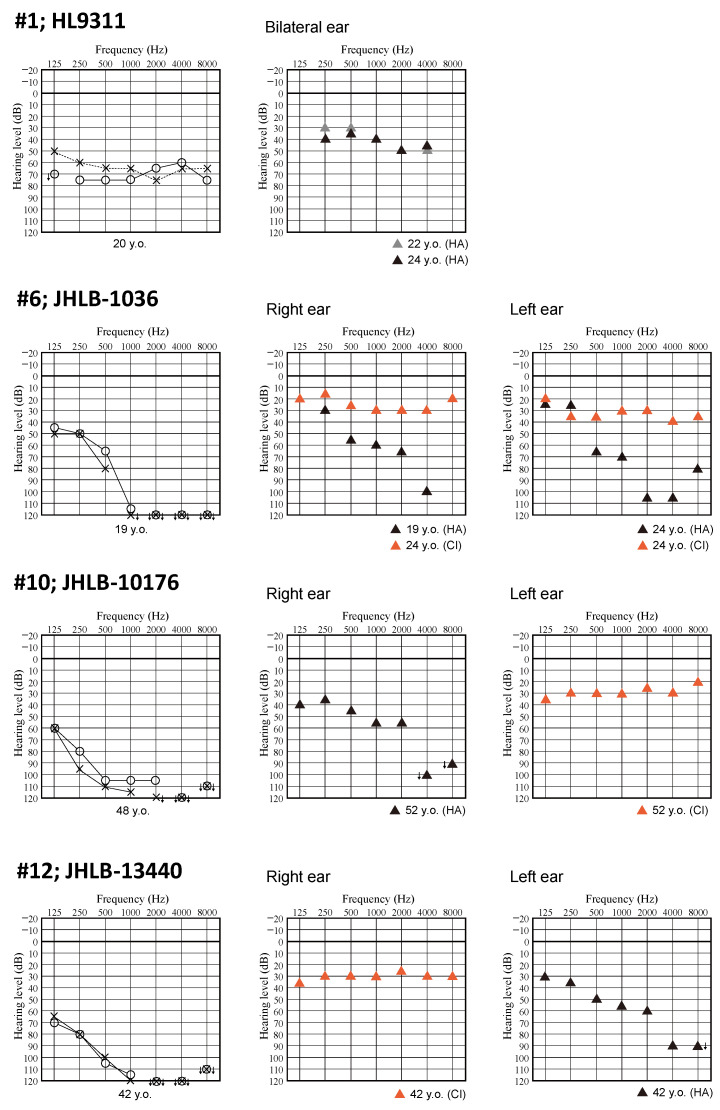
Audiograms and the hearing thresholds with hearing aid or cochlear implant. HA; hearing aid, CI; cochlear implant.

Figure 2 shows the time course of hearing level in two patients (family number 1 and 6). A female patient (family number 1) showed HL progression. Her hearing level deteriorated from 55 dB to 68.8 dB and 62.5 dB to 67.5 dB on PTA for the right and left ear, respectively, over 6 years. A male patient (family number 6) also showed HL progression. His hearing level deteriorated from 47.5 dB to 125 dB and 52.5 dB to 122.5 dB on PTA for the right and left ear, respectively, over 20 years. At the age of four, he had moderate HL and used bilateral hearing aids. The average sound field threshold level for patient #6 with hearing aids was about 35 dB. On progression to profound HL, he received bilateral CI. The postoperative average sound field threshold level with the cochlear implants was 32 dB in the right ear and 35 dB in the left ear (Figure 3). His postoperative Japanese speech discrimination scores measured 5 years after right cochlear implantation for monosyllables, words and sentences by iCI-2004 test (the Japanese speech discrimination scoring system) were 84, 100% and 100%, respectively. We were also able to obtain hearing thresholds with hearing devices including HAs or CI for four patients including this patient. The average sound field threshold level with the cochlear implant for three patients (family numbers 6, 10 and 12) was 30 dB (Figure 3).

## 4. Discussion

The *PTPRQ* gene encodes a member of the type III receptor-like protein tyrosine phosphatase family, and *PTPRQ* is known as one of the causative genes for non-syndromic SNHL without inner malformation. *PTPRQ*-associated HL is a relatively rare genetic cause of HL, with only about 30 causative *PTPRQ* variants reported to cause SNHL [7]. In this study, we identified 17 variants from 13 HL patients, which is the largest number of patients yet to be detected. The prevalence of *PTPRQ*-associated HL in this study was 0.08% (13/15,684). Sloan-Heggen et al. reported the NGS analysis results for 1119 American HL patients and, based on their report, the prevalence of *PTPRQ*-associated HL was 0.35% (4/1119) [26]. Abu Rayyan et al., reported that the prevalence of *PTPRQ*-associated HL in Palestinian patients was 0.61% (3/491) [27]. The prevalence varied among the study population, but all reports support the notion that *PTPRQ* was a rare causative gene for SNHL.

Among the 17 variants identified in this study, 15 (88.2%) were loss of function variants, with the majority of variants (16/25, 64%) previously reported as also being loss of function variants [23,28,29,30,31,32,33,34,35]. *PTPRQ* variants have been reported as causative for autosomal recessive and autosomal dominant HL [2]. However, all patients identified in this study carried biallelic variants and appeared to demonstrate autosomal recessive inheritance. In a previous report, the loss of function variants identified in the last coding exon (exon 45) were reported as causative for autosomal dominant HL (DFNA73). This variant resulted in the truncation of the protein by escaping nonsense-mediated mRNA decay and exerted a dominant-negative effect [23]. On the other hand, the loss of function variants identified in other exons were reported as causative for autosomal recessive HL (DFNB84) [7,23]. All variants identified in this study were located in exon 3 to exon 39 and no variants were located in exon 45. Thus, our findings were also consistent with those of previous reports.

With regard to the onset age, a majority of our patients (5/11, 45.4%) had congenital HL or prelingual onset HL (2/11, 18.2%). In addition, excluding only one case, all patients showed onset HL in their first decade. In previous reports, most patients with autosomal recessive inheritance also had congenital or prelingual HL [7,36,37,38,39,40,41,42]. Three cases of autosomal recessive *PTPRQ*-associated HL with post-lingual HL were reported in a single previous report (exact ages unknown) [28]. Thus, we concluded that the onset age for autosomal recessive *PTPRQ*-associated HL will be congenital or early onset within the first decade. With regard to the patients with autosomal dominant HL, the onset age was reported from two years to the fourth decade, with the majority being post-lingual (between the ages of 6 and 13) onset HL [23,43].

In this study, the hearing level at examination ranged from moderate to profound, with the HL progressive in all 11 patients for whom medical reports were available. The typical audiometric configuration of *PTPRQ*-associated HL was high-frequency steeply sloping or flat type. The hearing level of most patients with flat-type HL (6/8) was severe or profound. We obtained serial audiograms for two patients (family numbers 1 and 6) as shown in Figure 2. Patient #6 showed flat-type moderate HL at the age of four. His high frequency hearing then deteriorated from moderate to profound over 20 years, with his HL changing from flat-type to high-frequency steeply sloping HL. In previous studies, the hearing level in the patients with autosomal recessive inheritance ranged from moderate to profound [7,28,29,30,31,32,33,34,35]. Most patients had progressive, high-frequency HL regardless of autosomal recessive or autosomal dominant inheritance [7,23,28,37,38,39,40,41,42,43]. Three patients with flat-type severe HL were also reported [29,37]. Therefore, we concluded that the characteristic features of *PTPRQ*-associated HL involved the deterioration of high frequency hearing earlier than that at low frequency, with the HL progressing to severe or profound. Five patients received cochlear implantation and postoperative sound field threshold levels were favorable. Among these five patients, we could obtain Japanese monosyllable, words, and sentence discrimination scores for one patient with CI. This patient achieved a more favorable Japanese monosyllable discrimination score (84%) than the average of all CI patients assessed under the same test conditions (iCI-2004 test), as reported in our previous report (the average monosyllable discrimination score for 226 CI patients was 55.1 ± 19.6%) [44]. The outcomes of cochlear implantation for cases with *PTPRQ*-associated HL have not been reported previously. The *PTPRQ* protein localizes in the base of the stereocilia and is known to play crucial roles in maintaining the stereocilia structure [4,5,6]. As a result, HL induced by *PTPRQ* variants is caused by hair cell stereocilia degeneration, and this pathogenic mechanism supports the concept of beneficial outcomes for cochlear implantation. In our previous reports, we showed that the cochlear implantation outcomes for patients with an intra-cochlear etiology associated with a specific genetic background are potentially favorable [45,46]. The results of this study are consistent with those of our previous reports and also support this concept.

In this study, three patients reported symptoms of vertigo, while seven patients had no symptoms. A previous study revealed that hair bundles were lacking and vestibular evoked potentials were absent in *Ptprq* mutant mice [47]. Furthermore, it was reported that a Doberman Pinscher with a *PTPRQ* mutation had congenital HL and vestibular dysfunction [48]. Vestibular dysfunction in patients with *PTPRQ* variants was also demonstrated by video head impulse test (vHIT) [28]. However, other previous reports note that the results of neurotological examinations in patients with *PTPRQ*-associated HL were within the normal range [23,43]. Therefore, it remains unclear whether *PTPRQ* variants cause vestibular dysfunction in humans. Further studies including comprehensive vestibular assessments (caloric test, cervical vestibular evoked myogenic potential (cVEMP), ocular vestibular evoked myogenic potential (oVEMP) and vHIT) are required to estimate the role of *PTPRQ* in vestibular function in humans. In addition, *PTPRQ* gene expression in the human vestibule also remains unclear and further studies will be useful in estimating the role of *PTPRQ* in human vestibular function.

## 5. Conclusions

In conclusion, we identified 17 *PTPRQ* variants by analyzing over 15,000 Japanese patients with SNHL. Fifteen of the 17 variants were novel. The majority of variants identified in this study were loss of function. In most cases, HL onset was congenital or childhood. The hearing level at high frequency deteriorated earlier than that at low frequency, with the severity of HL progressing from moderate to severe or profound. Based on our results, there is a high likelihood that CI would be beneficial and is suggested for the profound HL patients with *PTPRQ*-associated HL. Further research is required to investigate the correlation between *PTPRQ* deficiency and its vestibular symptoms.

## Figures and Tables

**Table 1:** Clinical characteristics of the *PTPRQ*-associated hearing loss patients identified in this study.

Family Number	ID	Relationship	Base Change Allele 1	AA Change Allele 1	Base Change Allele 2	AA Change Allele 2	Hereditary	Onset	Age	Gender	Severity of HL	Type of HL	Progression	Vestibular Symptom	Hearing Device
1	HL9311	proband	c.279T>G	p.Y93 *	c.5270G>C	p.R1757T	sporadic	7	20	F	Moderate	Flat	Y	N	HA
2	JHLB-13162	proband	c.541delA	p.S181Afs * 12	c.5270G>C	p.R1757T	sporadic	0	18	F	Severe	Flat	Y	N	HA
3	JHLB-6375	proband	c.641C>A	p.S214 *	c.4093+1G>A	.	sporadic	0	34	F	Severe	HF steeply	Y	Y	CI
4	JHLB-2103	proband	c.745C>T	p.R249 *	c.5426+2T>C	.	sporadic	0	8	F	Severe	Flat	Y	N	HA
5	HL2182	proband	c.745C>T	p.R249 *	c.6017dupT	p.I2007Nfs * 14	NA	NA	NA	NA	NA	NA	NA	NA	
6	JHLB-1036	proband	c.745C>T	p.R249 *	c.745C>T	p.R249 *	sporadic	0	19	M	Profound	HF steeply	Y	N	CI
7	JHLB-12526	proband	c.1433_1436del	p.S479Kfs * 7	c.1433_1436del	p.S479Kfs * 7	AD	7	67	F	Profound	Flat	Y	NA	CI
8	JHLB12398	proband	c.1617delG	p.M539Ifs * 9	c.2030C>T	p.T677M	sporadic	13	29	M	Moderate	HF steeply	Y	Y	
9	JHLB-11894	proband	c.3206-2A>G	.	c.3263G>A	p.W1088 *	sporadic	3	37	F	Severe	HF steeply	Y	Y	HA
10	JHLB10176	proband	c.3263G>A	p.W1088 *	c.5270G>C	p.R1757T	sporadic	4	50	M	Profound	Flat	Y	N	CI
11	HL13084	proband	c.4093+1G>A	.	c.4402+1G>A	.	sporadic	6	27	M	Moderate	Flat	Y	N	
12	JHLB-13440	proband	c.4261G>T	p.E1421 *	c.4781_4783del	p.T1596del	sporadic	NA	42	F	Profound	Flat	NA	N	CI
13	O-1614	proband	c.4815C>A	p.Y1605 *	c.4815C>A	p.Y1605 *	sporadic	0	66	F	Profound	Flat	Y	NA	

* All variants are indicated on NM_001145026. NA: not available, HF: high frequency, Y: yes, N: no, HA: hearing aid, CI: cochlear implant.

**Table 2:** *PTPRQ* variants identified in this study.

Nucleotide Change	AA Change	Exon	PP2	MutTaster	REVEL	CADD	ToMMo 38KJPN	gnomAD All	Pathogenicity	Reference
c.279T>G	p.Y93 *	Exon 3	.	A	.	33	3.89 × 10^−5^	.	Pathogenic	This study
c.541delA	p.S181Afs * 12	Exon 5	.	.	.	.	.	.	Likely pathogenic	This study
c.641C>A	p.S214 *	Exon 5	.	A	.	39	.	.	Likely pathogenic	This study
c.745C>T	p.R249 *	Exon 6	.	A	.	37	9.03 × 10^−5^	1.95 × 10^−5^	Pathogenic	Sakuma et al., 2015 [24]
c.1433_1436del	p.S479Kfs * 7	Exon 10	.	.	.	.	.	.	Pathogenic	This study
c.1617delG	p.M539Ifs * 9	Exon 11	.	.	.	.	.	.	Likely pathogenic	This study
c.2030C>T	p.T677M	Exon 13	D	D	0.344	34	0.0005842	2.70 × 10^−5^	VUS	This study
c.3206-2A>G	.	Exon 20	.	D	.	22.8	.	.	Likely pathogenic	This study
c.3263G>A	p.W1088 *	Exon 20	.	A	.	40	2.59 × 10^−5^	6.55 × 10^−6^	Likely pathogenic	This study
c.4093+1G>A	.	Exon 23	.	D	.	25.5	6.46 × 10^−5^	6.58 × 10^−5^	Likely pathogenic	This study
c.4261G>T	p.E1421 *	Exon 25	.	A	.	54	.	.	Likely pathogenic	This study
c.4402+1G>A	.	Exon 25	.	D	.	27	0.0002614	.	Likely pathogenic	This study
c.4781_4783del	p.T1596del	Exon 28	.	.	.	.	7.79 × 10^−5^	6.53 × 10^−6^	VUS	This study
c.4815C>A	p.Y1605 *	Exon 28	.	A	.	38	1.30 × 10^−5^	.	Pathogenic	This study
c.5270G>C	p.R1757T	Exon 31	B	D	0.153	24.1	0.0002841	.	VUS	Yang et al., 2013 [25]
c.5426+2T>C	.	Exon 33	.	D	.	23.8	.	.	Likely pathogenic	This study
c.6017dupT	p.I2007Nfs * 14	Exon 39	.	.	.	.	.	.	Likely pathogenic	This study

* All variants are indicated on NM_001145026. AA: amino acid, PP2: PolyPhen-2, MutTaster: Mutation Taster, VUS: variant of uncertain significance.

## Data Availability

The datasets used during the current study are available from the corresponding author on reasonable request.
